# Glucocerebrosidase Mutations and Synucleinopathies. Potential Role of Sterylglucosides and Relevance of Studying Both GBA1 and GBA2 Genes

**DOI:** 10.3389/fnana.2018.00052

**Published:** 2018-06-28

**Authors:** Rafael Franco, Juan A. Sánchez-Arias, Gemma Navarro, José L. Lanciego

**Affiliations:** ^1^Department of Biochemistry and Molecular Biomedicine, School of Biology, University of Barcelona, Barcelona, Spain; ^2^Centro de Investigación Biomédica en Red de Enfermedades Neurodegenerativas (CiberNed), Instituto de Salud Carlos III, Madrid, Spain; ^3^Department of Neuroscience, Centro de Investigación Médica Aplicada (CIMA), University of Navarra, Pamplona, Spain; ^4^Department of Biochemistry and Physiology, School of Pharmacy, University of Barcelona, Barcelona, Spain; ^5^Department of Neuroscience, Instituto de Investigación Sanitaria de Navarra (IdiSNA), Pamplona, Spain

**Keywords:** GBA1, GCase, alpha-synuclein, lysosome, Parkinson’s disease, dementia with Lewy bodies, neurodegenerative disease

## Abstract

Gaucher’s disease (GD) is the most prevalent lysosomal storage disorder. GD is caused by homozygous mutations of the GBA1 gene, which codes for beta-glucocerebrosidase (GCase). Although GD primarily affects peripheral tissues, the presence of neurological symptoms has been reported in several GD subtypes. GBA1 mutations have recently deserved increased attention upon the demonstration that both homo- and heterozygous GBA1 mutations represent the most important genetic risk factor for the appearance of synucleinopathies like Parkinson’s disease (PD) and dementia with Lewy bodies (LBD). Although reduced GCase activity leads to alpha-synuclein aggregation, the mechanisms sustaining a role for GCase in alpha-synuclein homeostasis still remain largely unknown. Furthermore, the role to be played by impairment in the physiological function of endoplasmic reticulum, mitochondria and other subcellular membranous components is currently under investigation. Here we focus on the impact of GCase loss-of-function that impact on the levels of sterylglucosides, molecules that are known to trigger a PD-related synucleinopathy upon administration in rats. Moreover, the concurrence of another gene also coding for an enzyme with GCase activity (GBA2 gene) should also be taken into consideration, bearing in mind that in addition to a hydrolytic function, both GCases also share transglycosylation as a second catalytic activity. Accordingly, sterylglycoside levels should also be considered to further assess their impact on the neurodegenerative process. In this regard—and besides GBA1 genotyping—we suggest that screening for GBA2 mutations should be considered, together with analytical measurements of cholesterol glycosides in body fluids, as biomarkers for both PD risk and disease progression.

## Introduction

Glucocerebrosidases (GCases) catalyze the hydrolysis of D-glucosyl-N-acylsphingosine to D-glucose and N-acylsphingosine (E.C. enzyme entry: 3.2.1.45) and, therefore, the systematic name is D-glucosyl-N-acylsphingosine glucohydrolase. They may also catalyze the hydrolysis of other sugar derivatives, i.e., of Glycosyl-N-acylsphingosine to N-acylsphingosine and the corresponding sugar (E.C. enzyme entry: 3.2.1.62). Further enzyme activities have been described for GCases, thus showing that they have broad substrate specificity. For instance, according to the IUBMB enzyme nomenclature database^1^, they may display lactase activity (lactase hydrolysis to glucose and galactose; EC enzyme entry: 3.2.1.108).

**Figure 1:**
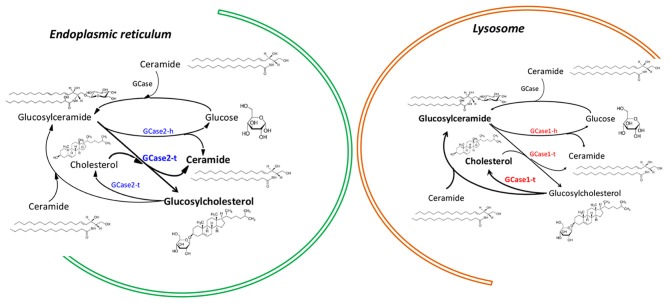
Differential action of enzymes encoded by GBA1 and GBA2 genes in the endoplasmic reticulum and in lysosomes. The nomenclature for GBA1-encoded and GBA-2-encoded enzymes is, respectively, GCase1 and GCase2. In both enzymes hydrolase activity is denoted with a terminal “h” and transferase activity with a terminal “t”.

GBA1 ß-glucosylceramidase gene is located in 1q22 of chromosome 1 of the human genome (Gene ID: 2629) and its gene product is a lysosomal enzyme with GCase activity. GBA2 ß-glucosylceramidase gene is located in 9p13.3 of chromosome 9 of the human genome (Gene ID: 57704, updated on Oct-2017) and also encodes for a protein with GCase activity. The product of the GBA2 gene was discovered long time ago by van Weely et al. (1993), who demonstrated that, unlike the firstly-described GCase, the product of the GBA2 gene was non-lysosomal, weakly associated to membranes and unrelated to Gaucher’s disease (GD; Figure 1). Identification of the gene and confirmation of the main characteristics of the gene products were achieved and reported years later by Boot et al. (2007).

## The Genetic Link Between GBA1 Gene Mutations and Synucleinopathies

Since a first case-report published in 1939 (Van Bogaert, 1939), more convincing evidence showing an association of GD and Parkinson’s disease (PD) was provided by Neudorfer et al. (1996), who reported six GD patients exhibiting “*typical extrapyramidal symptoms*” such as tremor, rigidity and bradykinesia. Later on, multicenter studies recruiting several thousands of patients revealed that GBA1 mutations are the most common genetic risk factor for developing PD (Sidransky et al., 2009; Velayati et al., 2010) In fact, a marked incidence of GBA1 mutations has been found in familial forms of PD (Mitsui et al., 2009; Nichols et al., 2009) as well as in up to 17% of PD patients that underwent deep brain stimulation (Angeli et al., 2013). Overall, it has been estimated that some GBA1 mutations may increase 20- to 30-fold the PD risk (Spitz et al., 2008; Sidransky et al., 2009; Bultron et al., 2010; McNeill et al., 2012; Mao et al., 2013; Blanz and Saftig, 2016; Migdalska-Richards and Schapira, 2016; Aflaki et al., 2017; O’Regan et al., 2017; Standaert, 2017). Moreover, the association of GBA1 mutations and dementia with Lewy bodies (LBD) is even stronger than for PD (Mata et al., 2008; Nalls et al., 2013). The association between GBA1 mutations and synucleinopathies other than PD and dementia with LBD, such as multiple system atrophy, still remains controversial (Asselta et al., 2014; Sklerov et al., 2017). A further interesting fact is that the type of mutations may increase the risk of one given synucleinopathy. In this regard and considering the two most frequent GBA1 mutations—L444P and N370S—the latter been regarded as more closely related to PD, whereas the L444P mutation seems to be more likely associated with LBD (Cilia et al., 2016). That said, it is also worth noting that PD and DLB share together a number of clinical and pathological features and indeed these clinical entities can even be considered as very close diseases (Friedman, 2018).

Regarding clinical phenotype, the onset occurs earlier in PD patients carrying a GBA1 mutation but the symptoms and efficacy of anti-PD medication is similar to those in idiopathic patients (Blanz and Saftig, 2016; Migdalska-Richards and Schapira, 2016; Aflaki et al., 2017). GBA1 mutation carriers are, however, more frequently affected by cognitive deficits and neuropsychiatric symptoms. Furthermore, the neuropathological fingerprint of Gaucher’s-associated PD is very similar to PD without GBA1 gene mutations, both conditions showing different age at death but no differences in “*total or regional semi-quantitative scores for Lewy-type synucleinopathy, senile plaques, neurofibrillary tangles, white matter rarefaction or cerebral amyloid angiopathy scores*” (Adler et al., 2017). Hence, it is tempting to speculate that GCase activity is a common underlying factor triggering alpha-synuclein-induced neurodegenerative processes in both GBA1 mutation carrier and non-carrier PD patients.

## GCase Gain- and Loss-of-Function Alternatives

The mechanisms by which GBA1 mutations could lead to PD were assessed by Velayati et al. (2010) who were, to our knowledge, the first to suggest that α-synuclein was at the center stage. The authors hypothesized that aggregation of α-synuclein was occurring either by “*enhanced protein aggregation or as a consequence of glucocerebrosidase deficiency*.” Authors also analyzed the two canonical alternative possibilities, i.e., that the disease could derive from a loss-of-function or a gain-of-function of GCase activity. Authors suggested that GCase gain-of function could explain deficits in proteostasis by the proteasome, endoplasmic reticulum stress and autophagy engagement. Since then, other authors have reinforced the idea that GBA1 mutations contribute to alpha-synuclein aggregation and engagement of autophagy and lysosome-mediated processes (GCase is a lysosomal enzyme; Westbroek et al., 2011; Beavan and Schapira, 2013; Dehay et al., 2013; Manzoni and Lewis, 2013; Pan and Yue, 2014; Zhang et al., 2014; Magalhaes et al., 2016; García-Sanz et al., 2018; Kinghorn et al., 2017).

It should be noted that congenital diseases involving enzymes are almost exclusively due to a loss of function (Scriver et al., 2001). After millions of years of evolution, it is not impossible but unlikely that a mutant enzyme leads to a gain of function, at the very least in what concerns the catalytic activity. In almost any inherited disease caused by mutations in the gene for any given enzyme, the activity is at the center stage and GCase is not an exception to this general rule. Indeed, it is broadly assumed that GD is due to reduced GCase activity, with a residual 32%–38% enzymatic activity accounting for GD type 1 (non-neuropathic GD), down to 13%–24% of reduced activity when considering neuropathic GD types 2 and 3 (Malini et al., 2014). Recent reports in the *Drosophila* model provide more complexity to the link between GCase activity and proteinopathies resulting in neuronal death. In one of them GCase deficient flies show protein aggregation and neurodegeneration but unrelated to α-synuclein (Davis et al., 2016). In the second, silencing the GBA gene affects locomotion by enhancing the aggregation of the PD-associated A73T α-synuclein gene mutant (Abul Khair et al., 2018).

In some cases, clinical symptoms appear only in homozygous states when GCase enzymatic activity is nearly zero. GD patients express mutant proteins displaying very diverse activity when measured as terms of GCase. On looking at clinical parameters from different patients it is assumed that the different GCase activity is the cause of the huge variety of symptoms involving different tissues and with varied intensity. For instance, the E326K PD-associated GCase1 variant (Berge-Seidl et al., 2017) may manifest GD subtypes 1, 2 or 3. It should be noted that there was controversy on whether it was a mutation or a polymorphism, as its presence in GD patients was accompanied with other GCase mutants (Park et al., 2002). Type 1 is characterized with the absence of neurological alterations, type 3 courses with signs of neurological disturbances and type 2 also courses with neurological alterations but severe and with early-appearing clinical symptoms, even at 3-months of age (Scriver et al., 2001; Horowitz et al., 2011). Being obvious that alterations of a lysosomal enzyme may influence the handling of proteins such as synucleins and, ultimately, engage autophagy, it is surely necessary to emit hypotheses aimed to better understand how a reduced enzyme activity may affect neuronal fate.

## Reduced GCase Activity May Lead to Neurotoxic Levels of Sterol Glycosides

Cholesterol and its derivatives are key molecules for the life of higher animal organisms. Cholesterol homeostasis is fundamental in a myriad of molecular processes ranging from membrane integrity to neurotransmission. Then, any alteration in the activity of the enzymes handling cholesterol should be taken into consideration. In this regard, while the presence of cholesterol has been widely studied, identification and distribution of cholesterol derivatives in subcellular compartments and, more importantly, of their function is largely incomplete (to date). Besides the classical neurotoxin-based rodent and primate models of PD (6-OHDA and MPTP), a new model has been recently developed. This model stands on the administration—to rats—of β-sitosterol β-D-glucoside (BSSG) leading to a progressive model of the disease. One of the many relevant features of the model is that, unlike other PD animal models, it leads to the accumulation of aggregated alpha-synuclein closely mimicking LBD (Van Kampen et al., 2015; Van Kampen and Robertson, 2017). These findings lead to hypothesize that accumulation of endogenous sterylglycosides may be neurotoxic for dopaminergic neurons. Reduced activity of GCase leads to increased levels of glucosylated lipids and of cholesterol and its esters (Magalhaes et al., 2016; Galvagnion, 2017). In fact, it has been recently found in fibroblasts from parkinsonian patients that the N370S-GBA1 mutation leads to lysosomal accumulation of cholesterol (García-Sanz et al., 2017). Also interesting is the recent elucidation of the structure of lipid-bound luminal domain of lysosomal integral membrane protein-2 (LIMP-2), which acts as a GCase receptor that allows trafficking of the enzyme from the endoplasmic reticulum to lysosomes, where it participates in sphingolipid processing (Reczek et al., 2007). Depending on the interaction with phospholipids and cholesterol, LIMP-2 shifts from a glucocerebrosidase-binding monomer to a dimer with differential lipid-exchange potential (Conrad et al., 2017).

It has been confirmed that GCase may display a second catalytic activity, namely glucosyltransferase (GCase1-t in Figure 1). Such chemical activity consists of transglucosylation, resulting in the transfer of the carbohydrate moiety from glucosylceramide (GlcCer) to cholesterol to form β-cholesteryl glucoside (GlcChol; Figure 1). Reversible transfer of a glucose moiety (transglucosylation) between GlcChol and GlcCer is catalyzed by GCase (Marques et al., 2016). Although the *in vivo* regulation of transglucosylation remains unknown, evidence shows that in non-pathological conditions GCase hydrolytic activity (GCase1-h in Figure 1) promotes the degradation of GlcChol. Mutant GCase could then be a factor promoting increased levels of GlcChol.

Analysis of sterylglucoside composition in the mammalian brain suggests a heterogeneous composition that includes the β-sitosterylglucoside (Akiyama and Hirabayashi, 2017). The above-described consequences of BSSG administration fits well with the finding that steryl-β-glucoside (ASG) contained in Cycad seeds directly enhances *in vitro* aggregation and cytotoxicity of alpha-synuclein (Van Kampen et al., 2015). Interestingly, inhabitants of Guam archipelago suffer from a severe syndrome apparently triggered by β-sitosterylglucoside accumulation after ingestion of Cycad plant-seed-derived products. The degenerative disease is known as ALS-PDC due to symptoms of amyotrophic lateral sclerosis and/or of the so-called parkinsonism/dementia complex (Shaw et al., 2002; Steele and McGeer, 2008). In keeping with neurotoxicity after consumption (Khabazian et al., 2002; Tabata et al., 2008), both neurotoxicity and links with PD have been confirmed by showing in rats that the synthetic β-sitosterylglucoside leads to a progressive parkinsonism whose clinical and histopathology scores may be sustained over the time and continue after cessation of the neurotoxic insult (Van Kampen et al., 2015). Due to the relatively poor specificity of GCase (see above) it is likely that the enzyme may use O-linked glycosides as substrates. Glucose binds to sitosterol in a similar manner that it binds to ceramide (Ivorra et al., 1988). Reduced GCase activity could then lead to accumulation of steryl-glycoside neurotoxic compounds.

## Both the β-Glucosidase Activity and Glucosyl Cholesterol Deserve Attention in PD

Regarding PD, the development in a rodent model of a progressive parkinsonian phenotype by β-sitosterylglucoside is in full keeping with the finding that ASG contained in Cycad seeds directly enhances the *in vitro* aggregation and cytotoxicity of alpha-synuclein (Van Kampen et al., 2015). By using electrospray ionization mass Spectrometry (ESI-MS/MS) assays, ß-glucosyl-cholesterol (GlcChol) has been detected in body fluids and tissues, including the central nervous system (CNS; Marques et al., 2016). Since the chromatographic behavior of galactosylceramide (GalCer), which is abundant in the CNS, is similar to that of GlcChol, it has been a technical challenge the assessment of GlcChol identification and quantitation in brain samples. There is no doubt now that GlcChol is present in the vertebrate brain. The compound is also detectable in blood plasma and, therefore, it is one of the parameters that might be followed in PD patients. It should be noted that (Marques et al., 2016) have reported that GlcChol plasma levels are higher in symptomatic GD patients.

Several *in vitro* examples of alterations due to unbalanced levels of sterol derivatives have been made available in the literature. For instance GlcChol regulates heat shock and stress response *in vitro* (Kunimoto et al., 2000, 2002). Exposure of fibroblasts to the compound induces rapid activation of heat shock transcription factor 1, binding to heat shock element and induction of heat shock protein 70, thus showing GlcChol as a significant mediator of stress responses. In *in vitro* cultures of NSC34 cells, which constitute a motoneuron cell model, low concentrations of GlcChol is neuroprotective whereas chronic exposure to GlcChol, i.e., in conditions resulting from defects on GCase-driven transglucosylation, results in neurotoxicity (Ly et al., 2008).

## GBA1 vs. GBA2 and Hydrolysis vs. Transglucosylation: The Crucial Balance to Regulate Steryl-Glucoside Levels

In the context of common features shared by GD and PD, hydrolysis has been the only activity that has been considered (Futerman and Hardy, 2016). GBA2 is a second gene that encodes a protein that, similarly to that encoded by GBA1 has two activities: hydrolysis (GCase) and the glucosyl transfer. The additional transglucosylation activity of enzymes coded by GBA1 and GBA2, their role in regulating the concentration of sterylglucosides and the different subcellular localization of the different GCases should be kept in mind to properly understand some conflicting data (Figure 1). Moreover, the complex regulation of cholesterol homeostasis regulation and its high individual variation could also explain the broad diversity of clinical symptoms that are shared by the GD and synucleinopathies.

Transglucosylation activity of the products of GBA genes has not yet attracted too much attention in what concerns to the potential link with origin and progression of both PD and GD. It is reasonable to hypothesize that hydrolysis (controlling ceramide and GlcCer levels), and transglucosylation (controlling GlcChol concentration and function) are both responsible of some of the clinical traits in GD, in particular those concerning the heterogeneity of symptoms.

How steryl-glucosides are synthesized? Paradoxically, different GCases (see below for details) are able to both hydrolyze and synthesize steryl-glucosides. Synthesis is achieved by transglucosylation and the short answer is that the enzyme, located in lysosomes, is likely to be more engaged in hydrolysis (Akiyama et al., 2011, 2013) whereas GBA2-coded GCase, which is unrelated to GBA1-coded GCase and not located in lysosomes, is more likely related to the formation of steryl-glucosides. The level of steryl-glucosides in a neuron depends on the balance between the hydrolytic and transglucosylation activities of these two different proteins, each being coded by one of two unrelated genes. In other words, sterylglucoside metabolism is controlled by both GBA1-coded GCase and by GBA2-coded GCase (see Akiyama et al., 2016; Akiyama and Hirabayashi, 2017). Hence: (i) the impact of mutations in the GBA1 gene that are linked to PD increased genetic risk should be studied in both hydrolysis and transglucosylation activities, and (ii) alterations/mutations in the GBA2 gene should be studied in parallel with alterations/mutations in the GBA1 gene.

In non-pathological steady-state conditions the GBA1 gene product promotes the degradation of GlcChol. In contrast, in similar conditions, the GBA2 gene product seems to work in the opposite direction (GlcChol synthesis). The balance keeping GlcChol levels within a narrow physiological range may by altered in pathological situations, even without mutations in the genes of the enzymes. For both proteins, transglucosylation is reversible and, accordingly, will proceed in one or another direction depending on availability of substrates/products. Given the different subcellular localization of GBA1 and GBA2 and fluctuations in sterols and sphingolipids that conceivably occur in healthy and altered neural cells, a disbalance on sphingolipids and sterol levels may occur. The scenario becomes more complex upon arrival of cholesterol from lipoproteins or release of ceramide from sphingomyelin. In those alternative events and in pathological scenarios GBA-gene-derived enzymes acquire more relevance.

## Mechanisms of Toxicity Induced by Steryl-Glucosides

The fact that the balance between GBA1-coded GCase and GBA2-coded GCase affects the level of glycosyl sterol derivatives opens several lines of analysis focused on explaining neurotoxicity underlying mechanisms. Here we highlight just one that has not been previously contemplated in relationship with GD-related PD.

The Pink1/parkin pathway, intended to maintain mitochondrial integrity, is modulated by Akt, a kinase whose activity is in turn regulated by GlcChol. There is another important issue related to the ever increasing concern on the association of mitochondrial dysfunction and neurodegenerative diseases in general and particularly when considering synucleinopathies (Cuadrado-Tejedor et al., 2013; Bose and Beal, 2016; Al Shahrani et al., 2017; Burbulla et al., 2017; Gao et al., 2017; Liu and Zhu, 2017; Pech and Verstreken, 2018; Rocha et al., 2018). The content of cholesterol in mitochondrial membranes is low, something consistent with the assumed evolutionary origins, namely symbiotic inclusion of bacteria (whose cholesterol content in negligible) into eukaryotic cells. Surely, mitochondria react to cholesterol-derivatives and it has been reported that GlcChol alters cell respiration and mitochondria-generated reactive oxygen species (ROS) and respiration (Panov et al., 2010; Shi et al., 2013). Although the underlying mechanisms are not known, the effects of GlcChol occur under active oxidation of succinate and this fact is relevant as it has been reported that the major source of ROS generation in neuronal mitochondria is associated with succinate handling by the electron transport chain (Panov et al., 2009). Combining a higher ROS production with elevated cholesterol contents in mitochondria could eventually result in higher levels of oxysterols that are contributors to the pathophysiology of PD and both potential biomarkers and therapeutic targets of the illness (Doria et al., 2016).

## Conclusions and Suggestions for Achieving Mechanistic Insights

The link between GBA1 gene mutations and PD is more likely due to a loss-of-function of GCase activity; loss-of-function is more usual in congenital diseases affecting enzyme genes and reduced enzyme activity the main cause of disease-promoting alterations. The level of sterylglucosides and in particular of GlcChol, which are toxic upon accumulation, are controlled by both hydrolysis and transglucosylation activities of both GBA1e and GBA2e gene products. Consequently, polymorphisms/mutations in the GBA2 gene should be studied in PD patients, especially in those already carrying GBA1 gene mutations. The transglucosylation activity of proteins encoded by mutated GBA genes should be determined. As mentioned above, the homeostatic control of synthesis and degradation of GlcChol is extremely relevant due to their multiple roles in different cell types and cell compartments. Transglucosylation may help to find the cause of disease-promoting alterations by GBA gene mutations. Complexities surrounding cholesterol homeostasis and patient-to-patient variation in GCase hydrolytic/transglucosylation activities could underlie the broad diversity of clinical symptoms in GD and the complex pattern of disease course and PD clinical manifestations in PD. Accordingly, it would be also desirable to measure GlcChol levels in plasma and in cerebrospinal fluid. To have a reference it would be also very relevant to measure GlcChol levels in plasma and in post-mortem neurological tissue of GD type 2 patients (those with sooner neurological manifestations and reduced life expectancy). A relatively high level of GlcChol in these patients could reinforce the hypothesis of the loss-of-function as main culprit and could confirm the role of glucosylated sterols in PD-associated neuropathology.

## Author Contributions

RF, JS-A, GN and JL wrote the original manuscript and approved the re-submitted version. RF and JS-A designed and prepared the figure.

## Conflict of Interest Statement

The authors declare that the research was conducted in the absence of any commercial or financial relationships that could be construed as a potential conflict of interest.

## Footnotes

^1^www.sbcs.qmul.ac.uk/iubmb/enzyme/
